# The Ocular and Gut Microbiome Axis in Understanding Glaucoma: A Systematic Review

**DOI:** 10.3390/jcm15031245

**Published:** 2026-02-04

**Authors:** Bruno Songel-Sanchis, Laura Morales-Fernández, Javier García-Bardera, Noemí Güemes-Villahoz, José María Martínez-de-la-Casa, Julián García-Feijoo

**Affiliations:** 1 Instituto de Investigación Sanitaria del Hospital Clínico San Carlos (IdISSC), 28040 Madrid, Spain; 2 Servicio de Oftalmología, Hospital Clínico San Carlos, Departamento de Inmunología, Oftalmología y Otorrinolaringología, Faculta de Medicina, Universidad Complutense de Madrid, 28040 Madrid, Spain

**Keywords:** microbiota, glaucoma, ocular surface, primary open angle glaucoma, pseudoexfoliation glaucoma

## Abstract

**Background:** Glaucoma is a neurodegenerative disease and the second leading cause of irreversible blindness in developed countries. It is characterized by progressive loss of retinal ganglion cells (RGCs) and optic nerve axons, leading to permanent vision impairment. Although elevated intraocular pressure (IOP) is the main recognized risk factor, recent evidence suggests that ocular and gut microbiota may play a significant role in the onset and progression of glaucoma. **Objectives**: This study aimed to characterize ocular and gut microbiota alterations in patients with different types of glaucoma. **Methods**: Five searches were conducted between June and September 2025 using selected keywords. A total of 121 articles were identified, of which 14 met the inclusion criteria following the PRISMA 2020 guidelines. **Results**: Findings indicate a Mendelian genetic predisposition influencing microbiota composition associated with glaucoma development. Patients treated with benzalkonium chloride (BAK) showed increased Gram-negative and *Alphaproteobacteria* on the ocular surface, along with enhanced lipopolysaccharide synthesis. Compared with controls, glaucoma patients exhibited reduced *Corynebacterium mastiditis* and *Actinobacteria* and increased *Firmicutes*, *Proteobacteria*, and *Verrucomicrobiota*. Dysbiosis was more pronounced in patients with concurrent dry eye disease, characterized by higher Gram-negative taxa and pro-inflammatory microbial activity. **Conclusions**: Significant differences in ocular and gut microbiota were observed between glaucoma patients and controls, as well as among glaucoma subtypes such as pseudoexfoliation and primary open-angle glaucoma. Age-related dysbiosis and epigenetic factors appear to contribute to disease development. Microbiota profiling may offer new opportunities for improved prediction, management, and treatment of glaucoma.

## 1. Introduction

Glaucoma includes a diverse group of chronic, progressive neurodegenerative eye disorders, all characterized by the irreversible loss of retinal ganglion cells (RGCs), thinning of the retinal nerve fiber layer (RNFL), and cupping of the optic disk. This disease represents the leading cause of irreversible blindness worldwide, currently affecting an estimated 70 to 95 million individuals globally [1].

Based on large-scale, population-based surveys, more than 3.5% of adults aged 40 to 80 years are affected by glaucoma. The most prevalent subtype is primary open-angle glaucoma (POAG), especially among individuals of European, Hispanic, and African descent, whereas primary angle-closure glaucoma occurs more frequently in Asian populations [2,3,4].

Glaucoma is clinically categorized based on anterior chamber angle anatomy into open-angle and angle-closure types. It is further classified etiologically as either primary, where no identifiable cause is found, or secondary, resulting from other ocular or systemic diseases, trauma, or medications.

Elevated intraocular pressure (IOP) remains the most significant and well-established risk factor for both open-angle and angle-closure glaucoma [5,6,7]. However, disease progression can also occur in individuals with normal or well-controlled IOP, particularly in cases of normal tension glaucoma (NTG), suggesting the involvement of additional mechanisms. Other important risk factors include increasing age, a positive family history, and myopia, which has been consistently associated with increased POAG risk.

Diagnosis is often delayed because early-stage glaucoma is typically asymptomatic. However, advances in diagnostic imaging have greatly improved early detection. Spectral domain optical coherence tomography (SD-OCT) is now widely used to noninvasively detect and monitor structural changes in the RNFL. SD-OCT provides reproducible, high-resolution (5 µm) measurements of peripapillary RNFL thickness (RNFL-T), allowing for early identification of disease progression [8].

Although a variety of medical and surgical treatments are available, all current therapies share the common goal of halting further optic nerve damage. To date, no treatment exists that can reverse the neurodegeneration of RGCs or repair damage to the optic nerve. The multifactorial nature of POAG has posed significant challenges in creating reliable in vitro or in vivo disease models, and the precise pathogenesis remains only partially understood.

Emerging research has pointed to several additional mechanisms contributing to glaucoma pathophysiology. Immunological factors, particularly involving T cell dysregulation, have been observed in NTG patients [9]. Studies have identified a lack of regulatory T cell (Treg) induction and imbalances between pro-inflammatory and anti-inflammatory T helper (Th) cell subsets. Mitochondrial dysfunction has also been implicated, with POAG patients showing increased mitochondrial DNA mutations and reduced mitochondrial respiratory function in peripheral blood samples [10].

Oxidative stress appears to play a significant role, as elevated levels of oxidative biomarkers have been consistently detected in glaucoma patients. These oxidative changes may contribute to damage in both the RGC layer and the optic nerve head. Furthermore, inflammatory changes have been documented in both human and animal models, supporting the involvement of immune-mediated damage in glaucoma progression [11].

Another rapidly evolving area of research is the role of the microbiome, particularly the gut-eye and gut–brain axes, in glaucoma. Disruptions in the gut and oral microbiota may compromise the integrity of epithelial barriers, allowing the translocation of pathogens, endotoxins, and pro-inflammatory molecules into systemic circulation. This can trigger systemic inflammation and potentially promote immune dysregulation and localized ocular inflammation. Notably, similar microbiota alterations have been reported in other neurodegenerative diseases, such as Parkinson’s disease, amyotrophic lateral sclerosis (ALS), and multiple sclerosis, which also show decreased levels of beneficial butyrate-producing bacteria [12,13].

Given the parallels in immune dysfunction and neurodegeneration, it is increasingly hypothesized that the gut microbiome and serum metabolites may play a significant role in the pathogenesis of glaucoma. This hypothesis opens the door to new avenues of research and therapeutic approaches aimed at modulating microbiota composition and restoring immune homeostasis in glaucoma patients.

Recent evidence suggests that alterations in ocular and gut microbiomes are associated with glaucoma and may reflect underlying immune and inflammatory changes rather than a direct causal mechanism. Current evidence supports an association between microbiome dysbiosis and glaucoma; however, causality cannot be inferred due to the predominance of cross-sectional and genetic association studies.

## 2. Methods

This systematic review was carried out in accordance with the Preferred Reporting Items for Systematic Reviews and Meta-Analyses (PRISMA) guidelines [14]. The PRISMA 2020 checklist was applied during the preparation of the review to guarantee adherence to the PRISMA 2020 statement. This checklist has also been provided as Supplementary Materials.

Five extensive literature searches were performed in the PubMed database between June and September 2025. The search strategy included the following terms: “Ocular Microbiota and Glaucoma”, “Gut Microbiota and Glaucoma”, “Ocular Surface Microbiota and Glaucoma”, “Ocular Microbiome and Glaucoma”, “Ocular Surface Microbiome and Glaucoma”.

The inclusion criteria comprised original research articles presenting data, characterization, and information on gut and ocular microbiota published between January 2018 and May 2025. Editorials, the literature reviews, animal studies without human data, conference abstracts, non-English publications, studies lacking patient-related information, and articles not directly related to the primary objective of this systematic review were excluded.

**Figure 1:**
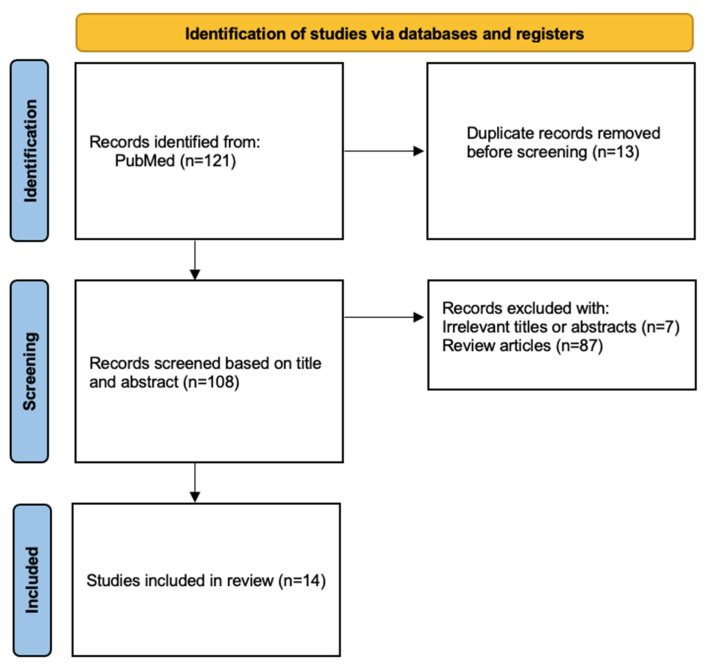
The PRISMA flow diagram for systematic reviews shows the process used to identify the papers included in the systematic review.

Following the searches, 121 articles were initially identified. Of these, 13 were excluded prior to screening due to duplicate entries, and 94 were removed for the following reasons: 7 articles were not related to the topic of interest or did not provide relevant data for the study, and 87 were bibliographic reviews. Consequently, 14 articles were ultimately included in the analysis (Figure 1).

The methodological quality of the studies included in this systematic review was assessed independently by three researchers (B.S.-S., L.M.-F., and J.G.-B.) using the Newcastle-Ottawa Scale (NOS) adapted for case–control studies. This scale assigns a score from 0 to 9 points across three domains: selection, comparability, and outcome. Based on the total score, studies were classified as poor quality (0–2 points), fair quality (3–5 points), and good/high quality (6–9 points) [15]. All the studies included in this systematic review achieved a score of 6 or higher, indicating good to high quality. The review protocol was not registered.

The included studies used heterogeneous methodologies for microbiome characterization. Ocular surface microbiota was primarily analyzed using 16S rRNA gene sequencing of conjunctival swabs, while gut microbiota was assessed using stool samples analyzed by 16S rRNA sequencing or inferred from genome-wide association study (GWAS) datasets in Mendelian randomization analyses. Proteomic and metabolomic assessments, when present, were reported as secondary outcomes of individual studies and were not systematically analyzed.

## 3. Results

The microbiome diversity in the ocular surface between control and glaucoma groups indicates that glaucoma and its treatment are associated with alterations in the ocular surface microbiome (OSM), frequently accompanied by clinical signs of ocular surface dysfunction.

In the Kamdougha et al. cohort study, 16S rRNA sequencing of 72 participants revealed that *Firmicutes* (Gram-positive, facultative anaerobes), *Proteobacteria* (Gram-negative, aerobes or facultative anaerobes), and *Bacteroidota* (Gram-negative, anaerobes) accounted for more than 98% of all conjunctival microbiota. Healthy controls demonstrated a predominance of *Actinobacteria* (Gram-positive, facultative anaerobes) (82,2%), whereas glaucoma patients, especially those with concomitant dry eye disease, presented a significant level of *Proteobacteria* and *Firmicutes* (*p* < 0.001 and *p* < 0.005, respectively). Increased alpha diversity (Table 1 and Table 2) (Shannon index) was reported only in the glaucoma and dry eye disease group (*p* = 0.05/0.2), while all patient groups differed significantly from controls in beta diversity (Bray–Curtis dissimilarity), with the strongest differences observed in the GDED group (*p* = 0.001 for GD and *p* = 0.01 for DED). Gram-negative taxa were elevated in pathological groups. Also, the glaucoma and dry eye disease group had the highest presence of inflammatory and stress-related bacterial functions.

Chang et al.’s study evaluated 68 subjects, comparing both treated and untreated eyes. A marked reduction in *Actinobacteriota* (80%) was observed in controls, while glaucoma eyes exhibited higher levels of *Firmicutes* (13%) and *Verrucomicrobiota* (12%). Patients with unilateral topical glaucoma therapy showed both treated and untreated glaucomatous eyes to harbor greater alpha diversity (*p* < 0.01), as beta diversity analysis also revealed substantial differences between groups (*p* < 0.01 for GD and DED) and increased abundance of Gram-negative organisms compared with controls. Microbiome alterations were correlated with reduced tear meniscus height and tear break-up time. Also, inferred pathways indicated increased lipopolysaccharide biosynthesis in glaucoma patients, consistent with heightened pro-inflammatory potential.

In contrast, Spörri et al. described slight taxonomic differences between glaucoma patients and controls. *Corynebacterium mastiditis* (Gram-positive, facultative anaerobe) is the only bacterium that shows a significant difference, being absent in all glaucoma cases but present in almost half of controls (44%). In addition to microbiome profiling, Spörri et al. reported tear proteomic alterations in glaucoma patients. These proteomic findings were analyzed independently from microbiome data and revealed differential expression of immune-related proteins, including complement activation pathways. No direct correlation analysis between microbial taxa and proteomic markers was performed (Table 1).

Across the selected studies, both microbiome- and proteome-based analyses revealed distinct microbial and molecular signatures differentiating glaucoma patients from healthy controls. In smaller 16S rRNA cohorts such as Gong et al. (2020) and Gong et al. (2022) [19,20], glaucoma groups showed enrichment of genera including *Megamonas*, *Bacteroides pelebeius*, *Prevotellaceae*, *Enterobacteriaceae*, and *Escherichia coli*, while reductions were observed for short-chain fatty acid–producing genera such as *Blautia* and *Fusicatenibacter*.

Larger population-based datasets, such as Zhou et al. (2024) [21] and Wu et al. (2024) [22], consistently identified higher relative abundance of families and genera, including *Victivallaceae*, *Lachnospiraceae*, *Lachnoclostridium*, *Oscillospira*, *Alloprevotella*, *Odoribacter*, *Eggerthella*, *Bilophila*, *Ruminoclostridium*, and *Lachnospiraceae UCG010*, with *p*-values ranging from 0.04 to 0.0003.

Metabolomic and proteomic studies further supported a distinctive glaucoma-associated biochemical profile: Skrzypecki et al. (2021) [23] reported significantly elevated trimethylamine levels (*p* = 0.001), whereas Chen et al. (2023) [24] found strong upregulation of inflammatory and immune-related proteins, including NFKB1, IL-18, KITLG, TLR9, FKBP2, and HDAC4 (all *p* < 0.02). Similarly, Wang et al. (2024) [25] documented increased levels of lactoferrin and α-1 antitrypsin in glaucoma cases (*p* = 0.01 and <0.001, respectively).

Genetic causal inference studies provided additional complementary evidence: Li & Lu (2023) [26] identified significant associations for *Eubacterium*, *Roseburia*, *Lachnospiraceae*, and *Ruminococcaceae* (all *p* ≈ 0.03–0.04), while J. Li et al. (2024) [27] demonstrated glaucoma-subtype-specific microbial patterns, including *Coprococcus 3*, *Ruminococcus gauvreauii group*, multiple members of *Erysipelotrichia*, *Anaerotruncus*, *Senegalimassilia*, and *Bacteroidota*, with strong statistical support (*p*-values 0.0003–0.01). In animal-based or mixed studies such as Zhang et al. (2022) [28], increases in *Romboutsia*, *Akkermansia*, *Bacteroides*, and markers of oxidative stress (GSH) were also noted. Finally, in Vergroesen et al. (2024) [29], several butyrate-producing taxa, such as *Butyrivibrio*, *Caproiciproducens*, *Clostridium sensu stricto 1*, *Coprococcus 1*, *Ruminococcaceae*, and *Shuttleworthia*, were consistently lower in glaucoma participants (Table 3).

Taken together, the aggregated evidence across cohorts, methodologies, and disease subtypes indicates a reproducible shift in glaucoma toward decreased beneficial commensal taxa and increased pro-inflammatory bacterial groups, accompanied by molecular alterations reflecting heightened immune activation.

## 4. Discussion

Benzalkonium chloride (BAK) and other preservatives are known to disrupt epithelial integrity and microbial homeostasis. Evidence from unilateral-treatment designs indicates that even untreated fellow eyes may be indirectly affected, possibly through systemic absorption, cross-contamination, or altered ocular surface [30,31]. The increased abundance of Gram-negative taxa, capable of producing pro-inflammatory molecules like lipopolysaccharide, may exacerbate ocular surface inflammation and contribute to dry eye manifestations. The lack of presence of *C. mastitidis* in glaucoma patients, a commensal associated with protective immune responses, raises the possibility that loss of beneficial microbes may predispose to dysregulated immunity. Currently, the glaucoma-specific tear proteomic profile, with increased immune proteins, underscores the predominance of host inflammatory responses over purely microbial alterations.

In the work by Spörri et al., a notable source of bias lies in the age imbalance between glaucoma patients and controls, as age is known to influence both the ocular microbiome and tear proteome, with the age-related dysbiosis [32]. Although multivariate analyses were performed to adjust for this factor, residual confounding cannot be fully excluded. Moreover, the relatively small sample size (*n* = 16 per group) may limit the statistical power and generalizability of the results.

Finally, the glaucoma patients were under multiple topical therapies, many of which are known to alter microbial communities, introducing potential treatment-related bias in the OSM composition [31].

In the study by Chang et al., the small cohort (10 patients, 7 controls) limits external validity and increases susceptibility to type I and II errors [33]. Additionally, all glaucoma therapies contained BAK, with its effects mentioned before. Another important limitation is the use of low-biomass samples [34], which can be prone to contamination despite negative controls. Furthermore, sequencing runs for patients and controls were performed separately, which raises the possibility of batch effects, although the authors reported no significant differences across runs.

Finally, Kamdougha et al. Despite a larger sample size compared to the previous studies, there was a gender imbalance, with women significantly overrepresented in the glaucoma with DED and DED-only groups [35]. This could bias microbial composition results, as sex is an established determinant of ocular and systemic microbiomes. Moreover, while groups were age-matched to controls, the glaucoma-only group was significantly older than the DED-only group, introducing age-related confounding. Another limitation is the reliance on the 16S rRNA sequencing, which, although informative, lacks the resolution of shotgun metagenomics and may misclassify taxa at lower levels [36]. The widespread use of preserved glaucoma eye drops in the patient groups also introduces treatment-related bias that may not be fully disentangled from disease-associated microbial changes.

Furthermore, host genetic factors may also influence baseline ocular surface microbiome composition, thus contributing to inter-subject variability and potentially modulating susceptibility to dysbiosis under disease or treatment stress. The concept of Mendelian predisposition implies that specific genetic variants could bias the microbial community toward certain taxa even before the onset of glaucoma or ocular surface disease. Although direct evidence linking inherited genetic variation to ocular surface microbiome structure in glaucoma is still lacking, analogous examples in other microbiome niches suggest that host genotype contributes a measurable fraction of microbiome variance [37].

Other methodological factors were found when interpreting these gut findings. First, all Mendelian Randomization (MR) studies relied on summary-level data derived from large population-based Genome-Wide Association Studies (GWAS), in which microbial composition was inferred from single-time-point stool samples; such measurements may poorly capture the dynamic and context-dependent nature of the gut microbiota [27].

Moreover, 16S rRNA sequencing remains limited in taxonomic resolution and is highly vulnerable to batch effects and contamination, an especially relevant concern in low-biomass microbiome studies [38]. Residual confounding may also persist if the genetic instruments for specific taxa influence glaucoma risk through pleiotropic immunometabolic pathways not fully accounted for by MR sensitivity analyses.

Additionally, MR approaches assume that host genetics explains a meaningful proportion of inter-individual microbiota variability, an assumption that remains imperfect, with heritability estimates for most taxa being low. The clinical cohorts underpinning these GWAS may differ substantially in diet, age, medication exposure, ethnicity, and environmental determinants of microbiota structure, introducing potential population-specific biases [39].

Finally, glaucoma itself is a heterogeneous disease, and MR analyses cannot distinguish between its diverse clinical subtypes or treatment exposures, both of which may independently affect microbial composition. These limitations emphasize the need for integrative, multi-omic, longitudinal studies to clarify the directionality, biological plausibility, and clinical relevance of microbiome-glaucoma associations.

Several limitations to this systematic review should be acknowledged. First, most included studies were cross-sectional or based on Mendelian randomization analyses, precluding causal inference regarding the role of microbiome alterations in glaucoma. Second, significant heterogeneity existed across studies in terms of design, sample size, microbiome assessment techniques, and patient characteristics, limiting direct comparability and generalizability. Third, most glaucoma patients were receiving topical treatments, frequently containing benzalkonium chloride, a known disruptor of epithelial integrity and microbial homeostasis, introducing treatment-related confounding. Fourth, ocular microbiome studies relied on low-biomass samples analyzed by 16S rRNA sequencing, which is susceptible to contamination and limited taxonomic resolution. Finally, Mendelian randomization studies were based on single-time-point microbiome measurements and assume a meaningful genetic contribution to microbiota variability, an assumption that remains only partially supported. These factors should be considered when interpreting the reported associations.

Additional studies are definitively needed in order to study the ocular surface and gut microbiome in glaucoma patients more in depth.

Despite these limitations, the transparent methodological approach and the clearly defined criteria used for study selection contribute to the consistency and reliability of this systematic review, offering a balanced and informative overview of the available evidence.

## 5. Conclusions

Overall, current evidence indicates consistent associations between glaucoma and alterations in ocular and gut microbiota. However, these findings should be interpreted cautiously, given the observational nature of most studies and potential treatment-related confounding. Future longitudinal and interventional studies are required to clarify the biological relevance and clinical implications of microbiome alterations in glaucoma.

## Figures and Tables

**Table 1:** Ocular surface microbiota in glaucoma and healthy control patients.

Author	Year	N Total	Control	Glaucoma	*p* Value
[16]	2025	72 (HC = 31, G = 13, GDED = 15, DED = 13)	-	*Firmicutes* PH (26%) GDED *Proteobacteria* PH (18%) G *Bacteroidota* PH (1%) GDED	<0.0001 <0.001 <0.005
[17]	2022	68 (HC = 28, GD = 20, G = 20)	*Actinobacteriota* PH (80%)	*Firmicutes* PH (13%) GD *Verrucomicrobiota* PH (12%) GD	<0.05 <0.05
[18]	2024	32 (HC = 16, G = 16)	*C.Mastiditis* SP (44%)	-	0.007

HC: healthy control. G: glaucoma. GDED: glaucoma with dry eye disease. DED: dry eye disease patients. GD: glaucoma treated with drops. PH: phylo. SP: species.

**Table 2:** Alpha and beta diversity in ocular surface microbiota in glaucoma and healthy control patients.

Author	Year	N Total	Shannon		Bray–Curtis		*p* Value
			Control	Glaucoma	Control	Glaucoma	
[16]	2025	72 (HC = 31, G = 13, GDED = 15, DED = 13)	HC - -	GDED - -	HC HC HC	GDED G DED	0.05/0.02 0.001 0.01
[17]	2022	68 (HC = 28, GD = 20, G = 20)	HC HC	GD G	HC HC	GD G	<0.01 <0.01
[18]	2024	32 (HC = 16, G = 16)	-	-	-	-	0.35

HC: healthy control. G: glaucoma. GDED: glaucoma with dry eye disease. DED: dry eye disease patients. GD: glaucoma treated with drops. For each case of significant difference in the Shannon and Bray—Curtis index, there was an increment for alpha and beta microbiome diversity, respectively, in the glaucoma group. The *p*-value is statistically significant at <0.05.

**Table 3:** Gut microbiome and proteome in glaucoma and healthy control patients.

Author	N Total	Control	Glaucoma	*p* Value
[19]	60 (HC = 30, G = 30)	*Megamonas* GN *Bacteroides pelebius* SP	*Prevotellaceae* FM *Enterobacteriaceae* GN *Escherichia coli* SP	-
[21]	224,431 (HC = 90.939, G = 133,492)	*Victivallaceae* FM *Lachnospiraceae* FM *Lachnoclostridium* GN *Oscillospira* GN *Alloprevotella* GN *Faecalibacterium* GN	*Euryarchaeota* PH *Odoribacter* GN *Ruminiclostridium* GN *Ruminococcaceae* FM/GN *Eubacterium* GN	0.04/0.003 0.03/0.04 0.02/0.002 0.01/0.02 0.02/0.03 0.04
[23]	40 (HC = 20, G = 20)	-	Trimethylamine	0.001
[24]	8 (HC = 4, G = 4)	-	NFKB1 IL18 KITLG TLR9 FKBP2 HDAC4	0.004 0.002 0.02 0.004 0.007 0.005
[22]	1.456.325 (HC = 717.854, G = 20.617)	*Oxalobacteraceae* FM *Eggerthella* GN	*Lachnospiraceae UCG010* GN *Bilophila* GN *Ruminiclostridium 9* GN	0.002/0.0003 0.003/0.001 0.003
[27]	372.540 (HC = 358.375, G = 7.756, GC = 1.199, XG = 3.125, NG = 2.085)	*Coprococcus3* GN (G) *Ruminiococcus gauvreauii group* GN (NG)	*Erysipelotrichia* CL (GC) *Erysipelotrichales* OD (GC) *Erysipelotrichaceae* FM (GC) *Anaerotruncus* GN (GC) *Senegalimassilia* GN (XG) *Bacteroidota* CL (XG) *Bacteroidales* OD (XG)	0.001/0.001 0.001/0.001 0.001 0.002 0.0003 0.01 0.01
[28]	-	*Lactobacillus* GN GSH	*Romboutsia* GN *Akkermansia* GN *Bacteroides* GN	0.04/0.05 0.01/0.004 0.01
[29]	1.472 (HC = 1.247, G = 225)	*Butyrivibrio* *Caproiciproducens* *Clostridium* *Coprococcus1* *Ruminococcaceae* *Shuttleworthia*	-	-
[25]	189 (HC = 75, G = 46, GC = 68)	-	Lactoferrin Alpha 1 antitrypsin	0.01 <0.001
[26]	456.944 (HC = 182.153, G = 104.973)	-	*Eubacterium* *Roseburia* *Lachnospiraceae* *Riminococcaceae*	0.04 0.03 0.03 0.03
[20]	60 (HC = 30, GC = 30)	*Blautia* *Fusicatenibacter*	-	-

HC: healthy control. G: primary open-angle glaucoma. GC: primary angle closure glaucoma. XG: exfoliation glaucoma. NG: normal tension glaucoma. PH: phylo. SP: species. FM: family. GN: genus. CL: class. OD: order.

## Data Availability

No new data were created or analyzed in this study.

## Supplementary Materials

The following supporting information can be downloaded at: https://www.mdpi.com/article/10.3390/jcm15031245/s1, Table S1: PRISMA 2020 checklist with all the items and their location in the systematic review.
